# Parent and Child Reports of Parenting Behaviors: Agreement Among a Longitudinal Study of Drug Court Participants

**DOI:** 10.3389/fpsyt.2021.667593

**Published:** 2021-06-29

**Authors:** Kate Guastaferro, Melissa C. Osborne, Betty S. Lai, Samantha S. Aubé, Wendy P. Guastaferro, Daniel J. Whitaker

**Affiliations:** ^1^Department of Human Development and Family Studies, College of Health and Human Development, The Pennsylvania State University, University Park, PA, United States; ^2^Byrdine F. Lewis College of Nursing and Health Professions, Georgia State University, Atlanta, GA, United States; ^3^Counseling, Developmental and Educational Psychology, Lynch School of Education and Human Development, Boston College, Boston, MA, United States; ^4^School of Criminology and Criminology, College of Social Work and Criminal Justice, Florida Atlantic University, Boca Raton, FL, United States; ^5^School of Public Health, Georgia State University, Atlanta, GA, United States

**Keywords:** concordance, parent-child relationship, parental substance use, mental health, drug court

## Abstract

Identifying ways to support children of parents with substance use disorder is a critical public health issue. This study focused on the parent-child relationship as a critical catalyst in child resilience. Using data from a longitudinal cohort study, the aims of this study were to: (1 ) examine the agreement between parent and child reports of parenting behaviors and (2 ) describe the association between agreement and child mental health. Participants were 50 parent-child dyads that included parents enrolled in an adult drug court and their children, aged 8–18. Overall, agreement (i.e., concordance) between parent and child reports of parenting was slight to fair. Parents reported their parenting behaviors to be slightly more positive than how children rated the same behaviors in the areas of: involvement, 0.53 (*SD* = 0.80); positive parenting, 0.66 (*SD* = 0.87), and monitoring behaviors, 0.46 (*SD* = 0.90). Parents also rated themselves, in comparison to their children's reports, as using less inconsistent discipline, −0.33 (*SD* = 1.00), and less corporal punishment, 0.13 (*SD* = 1.01). Agreement was related to some, but not all, child mental health outcomes. When parents rating their parenting as more positive than their child reported, that had a negative effect on child self-esteem and personal adjustment. Contrary to hypotheses, we did not find a significant relationship between positive parenting and internalizing problems. Findings have implications for obtaining parent and child reports of parenting within the drug court system, and for identifying children at higher risk for externalizing problems.

## Introduction

Identifying ways to support children of parents with substance use disorder is a critical public health issue. In the United States, about 8.7 million children, or 1 in 8, live in a home where at least one parent has a substance use disorder (1). Children of parents with a substance use disorder are at increased risk for life-long externalizing and internalizing behaviors (2–5). Parent substance use is a risk factor for poor parenting behaviors [e.g., lack of supervision, inconsistent discipline; (6, 7)] and child maltreatment (8, 9). In fact, parent substance use was documented among 29% of the 656,000 victims of child maltreatment in 2019 (10).

Adult drug courts are a community-based intervention for individuals with substance use disorder facing criminal charges. In the United States, there are more than 3,400 adult drug courts providing services to approximately 150,000 people each year (11, 12). Half of drug court participants in the United States are parents to at least one child under 18, one-fifth (20%) of whom are primary caregivers (13). The children of those individuals experience the negative effects of parental substance, as well as the negative effects of their parent's criminal justice involvement (14) and co-occurring parental mental health needs (15). It is known that a positive and strong parent-child relationship may mitigate negative outcomes for children (16). However, it is unknown if this holds true among drug court participant parents and their children, given the lack of information available about the parent-child relationship of drug court-involved parents (17).

Prior work has indicated that drug courts may provide a viable intervention point to address parenting and mental health needs and to improve the well-being of criminal justice-involved parents and their children (18). Yet, it is unclear how agencies (e.g., child welfare and criminal justice) and researchers may best identify issues or needs within the parent-child relationship. To date, it is unclear whether agencies should ask about parenting behaviors by querying the parent, the child, or both the parent and child. This paper addressed this issue by examining parent and child reports of parenting behaviors among a sample of families enrolled in an adult drug court (i.e., court mandated and monitored substance use treatment) in order to assess agreement and subsequent associations with child outcomes.

### Parent-Child Agreement on Parenting Behaviors

The degree of agreement (i.e., concordance) between two informants, a construct reflecting the extent to which informants share the same perspective on a domain (19), has been examined across a number of fields. In the parenting field, parents often underreport parenting problems compared to their children. For example, in a sample of 107 parent-child dyads parents indicated fewer parenting-related problems than their children (20). This is true of mothers and fathers. A study of 275 mother-father-child triads found that both mothers and fathers rated their parenting higher than their children's reports (21).

Beyond a problem of reliability or methodological error, a low level of agreement (i.e., discordance) between informants could potentially impact effectiveness studies and skew prevalence estimates (22). For example, in the child welfare field, examining the level of agreement between self-reported maltreatment and administrative data can allow for a better understanding of the prevalence of different types of maltreatment (e.g., physical abuse, sexual abuse, emotional abuse, and neglect). A systematic review of 13 articles found that youth tend to report more physical, sexual, and emotional abuse than found in casefiles, whereas casefiles indicate more neglect than reported by youth (23). Inaccurate accounts of maltreatment impact estimating prevalence and can impede the referral to much needed services or jeopardize child safety (24–28). It is important to understand the agreement between parent-child reports from an ethical perspective as well, as it is not always possible or feasible to assess or interview a child directly.

Further, it is unclear how parent-child agreement on perceived parenting behaviors predicts youth outcomes. Overall, parents generally rate parenting behaviors (e.g., monitoring, communication, discipline) more favorably than their child, indicating the parent believes they are engaging in positive parenting behaviors at a higher rate than their children experience them (29). A low degree of agreement between parents and children on parenting behaviors has been associated with negative child outcomes including substance use and poor psychological health (30–33). For example, in a study of 606 adolescents and their parents, significant discrepancies on parental monitoring related behaviors were associated with a greater likelihood of adolescent alcohol use (30). In a separate study of 484 adolescents and their parents, a high level of disagreement on parenting behavior (e.g., affection, control, punitiveness) was associated with high rates of adolescent reported anxiety and conduct disorder (33). Though we might expect there to be some level of disagreement between parent and child reports of parenting from a developmental perspective (34), it is important to understand the level of agreement, or lack thereof, and how it relates to child outcomes to best align preventative or treatment services to those in greatest need.

### Current Study

Children of substance using parents are a vulnerable population. However, perhaps because children of justice-involved parents are often overlooked by the criminal justice system (17), little is known about the parenting of drug court participants and the mental health needs of their children. Thus, the first aim of this study was to examine the degree of agreement between adult drug court parents and their child's report of parenting behaviors. Further, it is unknown how this degree of agreement may relate to subsequent child outcomes. To fill this void, we also sought to examine the extent to which agreement on parenting behaviors was associated with child mental health outcomes. The findings from the current study may be important in improving the efficiency and accuracy of assessment of need such that appropriate prevention services are made available to those in greatest need.

## Method

Data presented herein were extracted from a large quasi experimental longitudinal study of adult drug court parents in two Metro-Atlanta, Georgia felony-level programs. Funded by the Administration for Children and Families, the goal was to expand a regional partnership involving public and private partners across multiple systems to implement and evaluate evidence-based services to promote the well-being of children and family stability among individuals affected by substance abuse.

### Procedure and Participants

All procedures were approved by the Georgia State University Institutional Review Board. The original study design and procedures as well as a full description of the sample have been described in detail elsewhere [see (18)]. Briefly, data were collected from family units including the adult drug court participant, a child under 18 for whom they are a primary caregiver, and another caregiver of that child, if available. The assessment was administered via audio computer-assisted self-interview and overseen by a research assistant at the location of the participant's preference (e.g., home, treatment facility, library, coffee shop). Each participant who completed the assessment received a $75 gift card. Data were collected at enrollment (Time 1) and annually thereafter up to 3 years post enrollment.

The current analysis focuses on a subsample of 50 dyads of the drug court participant (hereafter called the “parent”) and child from Time 1 and Time 2 (1-year post enrollment). Participants were 50 parents and 50 matched children. Parents were predominantly male (58%), White (60%), and had a mean age of 38.2 years (*SD* = 7.76, range 25–54 years). Approximately half (47%) of parents reported an annual income < $25,000 and nearly one quarter of parents (24%) had less than a high school education. Participants were eligible if they identified as a primary caregiver of a child under 18, regardless of their biological relationship to the child. Nearly half of the parents (48%) reported they were married or living with a partner. Most (72%) identified as a biological parent of one of the children, others identified as stepparents (18%) and other relations (e.g., grandparent; 10%). Only one child participated per parent. The matched children were an average of 11.49 years old (*SD* = 2.70, range 8–18 years) and predominantly male (52%). Fifteen dyads were lost to attrition (e.g., either parent or child not available, child does not assent to continued participation). A sensitivity analysis indicated that there were no differences on baseline characteristics between those who completed assessments at both timepoints and those who completed only the baseline assessment.

### Measures

Sample characteristics (Time 1). Parents provided basic demographic characteristics at baseline including age, gender, income, educational attainment, and household constellation. Children reported age and gender. Parents also completed the Brief Child Abuse Potential Inventory [BCAP; (35)], an actuarial scale used to predict the potential for child maltreatment, at Time 1. Higher scores overall indicate an increased risk for maltreatment; a score ≧9 indicates the parent is at-risk for engaging in behaviors associated with maltreatment. Additionally, parents completed the Brief Symptom Inventory [BSI; (36)] to assess mental health across multiple domains (e.g., psychoticism, obsessive-compulsive, anxiety). Reported here are scores on the BSI General Severity Index (the overall severity based on the combination of symptoms and disruption of activities of daily life).

Parenting Behaviors (Time 1). Parents and children completed the Alabama Parenting Questionnaire (37) assessed at Time 1. The 35-item instrument assesses parental involvement (10 items; parent α = 0.80, child α = 0.84), positive parenting (6 items; parent α = 0.83, child α = 0.83), poor monitoring or supervision (9 items; parent α = 0.74, child α = 0.81), inconsistent discipline practices (6 items; parent α = 0.70, child α = 0.70), and corporal punishment (3 items; parent α = 0.65, child α = 0.76; 37). Seven other discipline practices (e.g., yelling, timeout) were summarized to provide greater detail about the parent-child relationship and to reduce implicit negative bias toward corporal punishment items (38). All 42 items are rated on a 5-point frequency scale (1= never to 5= always) with higher scores indicating a higher frequency of the behavior. As necessary, items were reverse-scored such that higher ratings indicate more favorable parenting behavior.

Child behavior outcomes (Time 2). The Behavior Assessment System for Children [BASC-2; (39)] measures positive and clinical dimensions of behavior and personality using multi-informant reports (i.e., child, parent, teacher). Children received age specific versions (e.g., ages 8–11 and 12–21) of the assessment of different lengths (e.g., 139 items vs. 176 items, respectively). Included in the present analysis are outcomes on 10 subscales reported at Time 2. Child self-report was preferred and was available for the internalizing problems, self-esteem, self-reliance, and personal adjustment subscales. Parent-reported data were used for the externalizing problems, adaptability, anxiety, leadership, social skills, and withdrawal subscales because child self-report was not available (i.e., these items were not answered by the child). Raw subscale scores were normed with the non-patient sample provided by the developer (39).

### Analytic Plan

Data were managed and analyzed using SAS version 9.4 (40). To measure the concordance between parent and child regarding parenting, weighted kappas and corresponding 95% confidence intervals (CI) were calculated for each item on the Alabama Parenting Questionnaire at baseline. The weighted kappa statistic takes into account that disagreement is not equally weighted among categories [e.g., a difference between 1 and 4 is given greater weight than a difference between 1 and 2; see (41)]. Kappa values range from−1 (indicating perfect disagreement) to 1 (indicating perfect agreement), with 0 indicating agreement due to chance. Strength of agreement was considered according to the standards put forth by Landis and Koch (42): 0–0.20 indicates slight agreement; 0.21–0.40 indicates fair agreement; 0.41–0.60 indicates moderate agreement; 0.61–0.80 indicates substantial agreement; and >0.81 indicates almost perfect agreement. Findings were considered statistically significant at the α = 0.05 level.

We then examined the association between the degree of difference between parent and child in each Alabama Parenting Questionnaire subscale (i.e., positive parenting, involvement, monitoring, inconsistent discipline, and corporal punishment) at Time 1 and child outcomes measured on the BASC-2 at Time 2. First, the degree of difference (hereafter referred to as the difference score) was quantified by subtracting the child response from the parent response for each Alabama Parenting Questionnaire item. Higher scores on this measure indicate more favorable parenting behavior. Thus, a positive difference score indicated that the parent rated the parenting behavior as being better than the child; a negative difference score indicated that the parent rated the parenting behavior as being worse than the child thought it was. Mean difference scores were then created for each Alabama Parenting Questionnaire subscale. These were calculated by taking the average of the difference scores for the items in that subscale. Next, we assessed the bivariate correlations between the Time 1 mean difference scores and the following child outcomes measured at Time 2: externalizing problems, internalizing problems, adaptability, self-esteem, self-reliance, anxiety, leadership, social skills, withdrawal, and personal adjustment. Bivariate correlations with a *p*-value of <0.10 were examined in separate multivariable linear regression models that controlled for Time 1 child age, parent education, and parent average score on all APQ items on the primary subscales. The decision to include these covariates was determined a priori based on theory and prior research. No other variables were included in the models in order to prevent them from becoming too complex, given the sample size (43). In the regression models, statistical significance was considered at the α = 0.05 level.

The analytic sample is comprised of 50 parent-child dyads who completed assessments at Time 1. Due to attrition, a total of 35 dyads completed both the Time 1 and Time 2 assessments. The percent and amount of missing data across study variables was as follows: 13.79% (*n* = 8) for child age, parent education, mean positive parenting difference score, and mean inconsistent discipline score at Time 1; 17.24% (*n* = 10) for mean corporal punishment difference score at baseline; and 25.86% (*n* = 15) for child self-esteem, self-reliance, and personal adjustment at Time 2. Missing data was addressed using listwise deletion. Additionally, one observation was removed from four of the regression models due to having both a high Cook's *d* and a studentized residual >3. Thus, the total *n* in the regression models ranged from 32 to 35.

## Results

At Time 1, the mean score on the BCAP was 6.10 (*SD* = 4.55); only 12 (24%) of the parents scored above the cut off of 9 indicating risk for maltreatment. The mean T-score on the BSI was 57.6 (*SD* = 11.79) indicating slightly increased mental health symptoms among parents. Self-reported child mental health outcomes as measured by the BASC-2 at Time 2 indicate that child self-esteem was near the average (T-score = 52.00; *SD* = 9.19) as was self-reliance (T-score = 50.29; *SD* = 10.29), and internalizing problems (T-score = 48.40; *SD* = 11.01). In contrast, personal adjustment (T-score = 38.54; *SD* = 7.77) was below average. Parent-reported child mental health outcomes at Time 2 indicate that child adaptability (T-score = 51.17; *SD* = 8.19), externalizing behaviors (T-score = 50.60; *SD* = 12.01), anxiety (T-score = 48.26; *SD* = 9.21), leadership (T-score = 51.17; *SD* = 10.01), social skills (T-score = 50.69; *SD* = 11.21), and withdrawal (T-score = 48.46; *SD* = 7.57) were near the average.

### Parent-Child Agreement on Parenting Behaviors

Overall, parents and children exhibited discrepancies in their ratings of parenting behaviors (Table 1). Mean difference in involvement score at Time 1 was 0.53 (*SD* = 0.80) indicating parents rated their involvement higher than children rated it (Table 2). Weighted kappas were statistically significant for three items on the involvement subscale. These ranged from 0.21 to 0.24, indicating fair agreement. Mean difference in positive parenting score at Time 1 was 0.66 (*SD* = 0.87), indicating that, on average, parents rated their parenting as being more positive than children thought it was. Weighted kappas were statistically significant for two items on the positive parenting subscale, with values of 0.13 and 0.16, indicating slight agreement. Mean difference in poor monitoring score at Time 1 was 0.46 (*SD* = 0.90). Weighted kappas were statistically significant for two items on the poor monitoring subscale, with values of 0.27 and 0.28, indicating fair agreement.

**Table 1:** Mean scores on Alabama Parenting Questionnaire subscales at baseline for parent-child dyads (*N* = 50).

**Subscales**	**Parents**	**Children**
	***M (SD)***	***M (SD)***
Corporal punishment	4.40 (1.63)	4.79 (2.63)
Inconsistent discipline	14.46 (4.28)	12.80 (5.28)
Other types of discipline	20.64 (2.94)	13.16 (5.29)
Poor monitoring/supervision	14.18 (4.62)	18.76 (8.40)
Positive parenting	26.80 (2.96)	22.85 (5.52)
Involvement	39.51 (5.40)	34.16 (9.04)

**Table 2:** Agreement between parent and child reports on parenting at baseline as measured by the Alabama Parenting Questionnaire (*N* = 50 dyads).

**Items by subscale**	**Parents**	**Children**	**Degree of difference**	**Weighted kappa**
	**Mean (SD)**	**Mean (SD)**	**Mean (SD)**	**95% CI**
**Involvement**				
You have a friendly talk with your child	4.38 (0.64)	3.74 (1.43)	1.22 (1.18)	0.05 [−0.09, 0.18]
You volunteer to help with special activities your child is involved in	3.58 (0.86)	3.29 (1.37)	1.00 (1.00)	0.24^*^ [0.06, 0.43]
You play games or do other fun things with your child	4.10 (0.76)	3.78 (1.16)	1.04 (0.82)	0.05 [−0.12, 0.22]
You ask your child about his/her day in school	4.52 (0.71)	4.14 (1.38)	0.80 (1.14)	0.23^*^ [0.05, 0.40]
You help your child with his/her homework	3.82 (0.94)	3.61 (1.32)	1.00 (0.82)	0.21^*^ [0.04, 0.39]
You ask your child what his/her plans are for the coming day	3.86 (0.95)	3.45 (1.39)	1.20 (0.98)	0.11 [−0.05, 0.27]
You drive your child to a special activity	3.92 (0.92)	3.51 (1.39)	1.22 (0.90)	0.08 [−0.08, 0.24]
You talk to your child about his/her friends	3.98 (0.94)	2.80 (1.55)	1.65 (1.16)	0.07 [−0.06, 0.20]
Your child helps plan family activities	3.78 (0.86)	3.42 (1.34)	1.24 (1.00)	0.03 [−0.12, 0.18]
You attend PTA meetings, parent/teacher conferences, or other meetings at your child's school	3.55 (1.31)	2.70 (1.53)	1.61 (1.16)	0.09 [−0.06, 0.23]
**Positive parenting**				
You let your child know when he/she is doing a good job with something	4.72 (0.54)	4.04 (1.23)	0.92 (1.08)	0.06 [−0.09, 0.21]
You reward or give something extra to your child for behaving well	4.04 (0.81)	3.56 (1.23)	1.12 (0.94)	0.06 [−0.10, 0.22]
You compliment your child when he/she does something well	4.68 (0.55)	4.08 (1.24)	0.84 (0.96)	0.13 [−0.03, 0.28]
You praise your child if he/she behaves well	4.54 (0.65)	3.84 (1.28)	0.96 (1.04)	0.16^*^ [0.02, 0.31]
You hug or kiss your child when he/she has done something well	4.48 (0.71)	3.50 (1.34)	1.18 (1.10)	0.13^*^ [0.01, 0.25]
You tell your child that you like it when he/she helps out around the house	4.34 (0.72)	3.86 (1.24)	0.98 (1.07)	0.11 [−0.07, 0.30]
**Poor monitoring/Supervision**				
Your child fails to leave a note or to let you know where he/she is going	1.60 (0.95)	1.88 (1.30)	0.96 (1.11)	0.11 [−0.08, 0.31]
Your child stays out in the evening past the time he/she is supposed to be home	1.48 (0.91)	1.58 (1.16)	0.70 (1.09)	0.19 [−0.04, 0.42]
Your child is out with friends you don't know.	1.28 (0.61)	2.39 (1.53)	1.39 (1.46)	−0.01 [−0.12, 0.11]
Child goes out without a curfew	1.26 (0.72)	2.32 (1.54)	1.30 (1.47)	0.04 [−0.06, 0.14]
Your child is out after dark without an adult with him/her	1.46 (0.93)	1.82 (1.33)	0.92 (1.34)	0.10 [−0.13, 0.34]
You get so busy that you forget where your child is and what he/she is doing	1.26 (0.63)	1.71 (1.22)	0.71 (1.11)	0.14 [−0.11, 0.39]
You don't check that your child comes home at the time he/she was supposed to	1.32 (0.87)	1.47 (1.12)	0.64 (1.24)	0.10 [−0.09, 0.28]
You don't tell your child where you are going	1.76 (1.06)	1.80 (1.26)	0.80 (1.06)	0.28^*^ [0.10, 0.46]
Your child comes home from school more than an hour past the time you expect him/her	1.16 (0.37)	1.47 (1.00)	0.46 (0.97)	0.12 [−0.08, 0.32]
You spank your child with your hand when he/she has done something wrong	1.58 (0.81)	2.31 (1.23)	0.90 (1.01)	0.27^*^ [0.10, 0.43]
**Inconsistent discipline**				
You threaten to punish your child and then do not actually punish him/her	3.18 (0.96)	2.12 (1.42)	1.55 (0.94)	0.10 [−0.02, 0.21]
Your child talks you out of being punished after he/she has done something wrong	2.38 (1.09)	2.10 (1.36)	1.16 (1.06)	0.16 [−0.03, 0.35]
You feel that getting your child to obey you is more trouble than it's worth	1.66 (1.06)	1.60 (1.27)	0.92 (1.37)	0.11 [−0.11, 0.32]
You let your child out of punishment early	2.84 (1.09)	2.63 (1.47)	1.29 (1.03)	0.13 [−0.06, 0.32]
Your child is not punished when he/she has done something wrong	2.24 (1.00)	2.15 (1.30)	1.27 (1.07)	−0.002 [−0.18. 0.17]
The punishment you give your child depends on your mood	2.16 (1.11)	2.21 (1.34)	1.15 (1.15)	0.13 [−0.06, 0.32]
**Corporal punishment**				
You spank your child with your hand when he/she has done something wrong	1.86 (0.90)	1.71 (1.17)	0.81 (1.00)	0.24^*^ [0.03, 0.45]
You slap your child when he/she has done something wrong	1.14 (0.45)	1.49 (0.98)	0.64 (1.01)	−0.12 [−0.20, −0.03]
You yell or scream at your child when he/she has done something wrong	2.54 (1.05)	2.06 (1.28)	1.49 (1.06)	−0.08 [−0.24, 0.08]

* *p < 0.05*.

Mean difference in inconsistent discipline score at Time 1 was −0.33 (*SD* = 1.00), indicating that parents thought they were less consistent with discipline than children rated them as being. None of the weighted kappas on this subscale reached statistical significance. Finally, mean difference in corporal punishment score at Time 1 was 0.13 (*SD* = 1.01), indicating that, on average, parents rated themselves as using less corporal punishment than children rated them as using. Weighted kappa for one item on the corporal punishment subscale was statistically significant, with a value of 0.24, indicating fair agreement. Note that there was substantial variability in each of these mean difference scores across the subscales, as indicated by the large standard deviations.

### Relationship Between Parent-Child Agreement and Child Mental Health Outcomes

Bivariate correlations between the parenting subscales (i.e., positive parenting, involvement, poor monitoring, inconsistent discipline, corporal punishment) and child mental health outcomes (i.e., subscales of the BASC-2) are reported in Table 3. There were significant correlations between mean positive parenting difference score and the child mental health outcomes of self-esteem, self-reliance, and internalizing problems (*p* < 0.10). In each case, the direction of the correlation indicated that the more the parents overestimated their positive parenting, the poorer the children's mental health (lower self-esteem, higher internalizing behaviors). There was also significant correlation between mean inconsistent discipline difference score and child personal adjustment. There were significant relationships between mean corporal punishment difference and self-esteem and personal adjustment subscales (*p* < 0.10). Greater parental overestimation of corporal punishment was related to lower self-esteem and adjustment. There were no significant correlations between mean difference scores on the involvement or monitoring subscales and the child mental health outcomes (*p* > 0.10).

**Table 3:** Bivariate correlations between mean positive parenting difference scores and child mental health outcomes.

**Mean difference score**	**Externalizing**	**Internalizing**	**Adaptability**	**Anxiety**	**Leadership**	**Social skills**	**Withdrawal**	**Self-esteem**	**Self-reliance**	**Personal adjustment**
**Child mental health subscales**										
Positive parenting	0.06	**0.31**	−0.01	−0.19	−0.10	−0.05	−0.01	**−0.33**	**−0.33**	−0.26
Involvement	−0.03	0.19	0.23	−0.21	0.19	0.08	−0.16	−0.22	−0.21	−0.10
Monitoring	−0.002	0.11	−0.16	−0.26	−0.13	−0.24	−0.13	−0.08	−0.18	−0.27
Inconsistent discipline	−0.09	0.07	−0.05	−0.18	0.07	−0.14	−0.21	−0.24	−0.25	**−0.33**
Corporal punishment	−0.003	0.03	0.03	0.03	0.07	−0.06	0.13	**−0.33**	−0.28	**−0.52**

*Numbers in bold indicate p < 0.10*.

All significant bivariate correlations were examined in separate multivariable linear regression models controlling for child age and parent education (Table 4). There was a statistically significant relationship between mean positive parenting difference score and child self-esteem at Time 2 (*B*=-3.87, 95% CI: −6.60, −1.15; *p* = 0.01), with higher difference scores (i.e., parent rating parenting as more positive than children) corresponding to lower levels of self-esteem. This indicates that the more parents rated their parenting as better than their children at Time 1, the lower children's self-esteem was at Time 2, on average. The models examining positive parenting and child self-reliance and internalizing problems did not yield statistically significant results.

**Table 4:** Multivariable linear regression models examining relationship between mean positive parenting difference score and child mental health outcomes.

**Variable**	**Model *n***	**Model Adj. *R^**2**^***	**Parameter estimate (*95% CI*)**	***p***	**Standardized estimate**
**Mean difference in positive parenting**					
Self-Esteem	34^*^	0.18	−3.87 [−6.60, −1.15]	0.007	−0.48
Self-reliance	34^*^	0.09	−2.82 [−6.18, 0.53]	0.096	−0.30
Internalizing problems	34^*^	0.07	0.27 [−3.45, 3.98]	0.884	0.03
**Mean difference in inconsistent discipline**					
Personal adjustment	35	0.09	−1.85 [−4.85, 1.14]	0.216	−0.23
**Mean difference in corporal punishment**					
Self-Esteem	32^*^	0.06	−2.84 [−5.79, 0.11]	0.058	−0.40
Personal adjustment	33	0.22	−3.24 [−5.97, −0.51]	0.022	−0.44

* *Removed 1 observation due to high Cook's d and studentized residual in these models; Models were adjusted for child age and parent education*.

The relationship between mean inconsistent discipline difference score and personal adjustment was not statistically significant (*B* = −1.85, 95% CI: −4.85, 1.14, *p* = 0.09), indicating that there is no evidence in this sample that a difference between parent and child with regard to inconsistent discipline is associated with child personal adjustment. Separate multivariable linear regression models examined the relationships between mean corporal punishment difference score and self-esteem and personal adjustment, respectively. There was a statistically significant association between mean corporal punishment difference score and personal adjustment (*B* = −3.24, 95% CI: −5.97, −0.51, *p* = 0.02), with higher difference scores (i.e., parents rating themselves as using corporal punishment less frequently than children did) corresponding to lower personal adjustment scores. This indicates that the more parents thought they were using less corporal punishment at Time 1 than their children did, the lower child personal adjustment was at Time 2.

## Discussion

The parent-child relationship may buffer against negative outcomes for children and launch children toward positive outcomes [e.g., favorable mental health; (16)]. This study evaluated agreement between parent and child reports of parenting among an at-risk population, families in the drug court system. Overall, in this sample the level of agreement between parent and child reports of parenting was slight to fair. Parents rated their parenting behaviors more favorably than did their children. Specifically, parents reported their involvement, positive parenting, and monitoring behaviors to be greater than what their children reported. These findings align with the broader literature (20, 21). In this sample, parents rated themselves as using less corporal punishment and less inconsistent discipline than children. This is aligned with prior research. For example, mother-child dyads from the Fragile Families and Child Well-being Study (*N* = 1,180), children reported a significantly higher rate of corporal punishment (i.e., spanking or physical punishment more than 10 times per year) than their mothers, 15–7%, respectively (44).

Results also suggest that the degree of agreement matters. Child self-reported self-esteem and personal adjustment was negatively affected when parents assessed their parenting more favorably than the child. However, this relationship was not found between positive parenting and internalizing problems, which went against hypotheses. Some prior research suggests when there is discordance among parent-adolescent reports of parenting, specifically affection, children report greater levels of anxiety (33). Other research indicates that parent-child disagreement has stronger associations with youth total problems than for youth internalizing problems (45).

While some disagreement is expected and found in previous literature, parent reporting of a higher quality relationship and greater monitoring (than youth reports) can influence negative developmental consequences for children. Rusby et al. (46) found discordance between youth and parents regarding their relationship and parental monitoring. In their study of eighth graders and their parents, parents significantly rated their relationship quality and monitoring higher than adolescents did and further, youth perceptions of parenting behaviors had an impact on youth substance use initiation. A meta-analysis of parent-adolescent conflict, disagreement, hostility, and youth maladjustment found parent-adolescent disagreement influenced negative outcomes in youth (45). Thus, assessing both parent and youth perspectives on parenting behaviors is important and merits continued study. The research presented here is novel in that the parents have an identified substance use disorder and are involved with the justice system because of their substance use.

Although this study has many important strengths, some limitations should be considered carefully. First, the sample, recruited from families involved in the drug court system, is modest in size. Although this is a major strength of the study, with regard to recruiting a vulnerable, at-risk sample, findings may not generalize. Related, there was 30% attrition rate from Time 1 to Time 2 among parent-child dyads with complete data. This is not unusual, but limits the conclusions made from these findings. In addition, all measures were self-reported. Child mental health outcomes reported by children and by parents were both within the normal range. While self-report is widely used and reflects what happens in the field, future studies might explore other factors related to other supports that ensured resilience. Additionally, future studies might include clinical interviews, observational measurement, or added respondents (i.e., schoolteacher reports) to bolster findings and draw more reliable conclusions. Finally, we acknowledge that alternative explanations for child mental health outcomes exist, and the inclusion of a different set of covariates may also have yielded different results. However, this study focused on the concordance between parents and children in their view of the parent-child relationship and its association with child mental health outcomes. Such outcomes may also be influenced by child maltreatment history, family history of mental health problems, previous mental health status, and other factors that were not measured in this study. Future research should include child's previous mental health status and consider other factors that may determine child mental health.

## Conclusion

Though the relationship between parental substance use and poor child health outcomes are well-established (4, 8, 9, 45), little is known about the level of agreement between parent and child reports of parenting. This paper makes a unique contribution by demonstrating slight to fair agreement between parent and child reports. Future research must examine if parent-child discrepancies in parenting in this population are larger or different than non-substance using parents. The answer has implication for treatment and measurement of parenting/child outcomes among parents with substance use disorders, whether parents are involved in the justice or child welfare system.

## Data Availability Statement

The data analyzed in this study is subject to the following licenses/restrictions: Data available upon reasonable request. Requests to access these data should be directed to dwhitaker@gsu.edu.

## Ethics Statement

The studies involving human participants were reviewed and approved by Georgia State University. Written informed consent to participate in this study was provided by the participants' legal guardian/next of kin.

## Author Contributions

The study was conceptualized by KG, MO, BL, WG, and DW. Analyses were completed by MO and reviewed by BL and KG. All authors contributed to writing sections of this manuscript and approve final manuscript.

## Conflict of Interest

The authors declare that the research was conducted in the absence of any commercial or financial relationships that could be construed as a potential conflict of interest.

## References

[1] Lipari RN Van Horn SL. Children Living with Parents Who Have a Substance Use Disorder (The CBHSQ Report). Center for Behavioral Health Statistics and Quality, Substance Abuse and Mental Health Services Administration (2017). Available online at: https://www.samhsa.gov/data/sites/default/files/report_3223/ShortReport-3223.html. 29144715

[2] Bountress K Chassin L. Risk for behavior problems in children of parents with substance use disorders. Am J Orthopsychiatry. (2015) 85:275–86. 10.1037/ort0000063 25985113 PMC4443848

[3] Clark DB Cornelius J Wood DS Vanyukov M. Psychopathology risk transmission in children of parents with substance use disorders. Am J Orthopsychiatr. (2004) 161:685–91. 10.1176/appi.ajp.161.4.685 15056515 PMC3155941

[4] Kelley ML Bravo AJ Hamrick HC Braitman AL White TD Jenkins J. Parents' reports of children's internalizing symptoms: associations with parents' mental health symptoms and substance use disorder. J Child Fam Stud. (2017) 26:1646–54. 10.1007/s10826-017-0677-9 29430165 PMC5800750

[5] Marmorstein NR Iacono WG McGue M. Alcohol and illicit drug dependence among parents: associations with offspring externalizing disorders. Psychol Med. (2009) 39:149–55. 10.1017/S0033291708003085 18410699 PMC2680685

[6] Calhoun S Conner E Miller M Messina N. Improving the outcomes of children affected by parental substance abuse: a review of randomized controlled trials. Subst Abuse Rehabil. (2015) 6:15–24. 10.2147/SAR.S46439 25670915 PMC4315541

[7] Fals-Stewart W Kelley ML Fincham FD Golden J Logsdon T. Emotional and behavioral problems of children living with drug-abusing fathers: comparisons with children living with alcohol-abusing and non-substance-abusing fathers. J Fam Psychol. (2004) 18:319–30. 10.1037/0893-3200.18.2.319 15222839

[8] Choi S Huang H Ryan JP. Substance abuse treatment completion in child welfare: does substance abuse treatment completion matter in the decision to reunify families? Child Youth Serv Rev. (2012) 34:1639–45. 10.1016/j.childyouth.2012.04.022

[9] Dube SR Anda RF Felitti VJ Croft JB Edwards VJ Giles WH. Growing up with parental alcohol abuse: exposure to childhood abuse, neglect, household dysfunction. Child Abuse Negl. (2001) 25:1627–40. 10.1016/S0145-2134(01)00293-9 11814159

[10] DHHS. Child Maltreatment 2019. U.S. Department of Health and Human Services, Administration for Children and Families, Administration on Children, Youth and Families, Children's Bureau. https://www.acf.hhs.gov/cb/research-data-technology/statistics-research/child-maltreatment (2021).

[11] NADCP. A Historic Year for NADCP and Treatment Courts! - NADCP.org. NADCP National Association of Drug Court Professionals. (2018). Available online at: https://www.nadcp.org/a-historic-year-for-nadcp-and-treatment-courts/.

[12] NDCRC. What are Drug Courts? National Drug Court Resource Center. Available online at: https://ndcrc.org/what-are-drug-courts (2018).

[13] Rossman SB Roman JK Zweig JM Lindquist CH Rempel M Willison JB. The Multi-Site Adult Drug Court Evaluation: Study Overview and Design, Volume 1 (No. 237109). Urban Institute Justice Policy Center (2011). Available online at: https://www.ncjrs.gov/pdffiles1/nij/grants/237109.pdf 10.1037/e718342011-001

[14] Turanovic JJ Rodriguez N Pratt TC. The collateral consequences of incarceration revisited: a qualitative analysis of the effects on caregivers of children of incarcerated parents^*^. Criminology. (2012) 50:913–59. 10.1111/j.1745-9125.2012.00283.x

[15] Osher F D'Amora DA Plotkin M Jarrett N Eggleston A. Adults with Behavioral Health Needs under Correctional Supervision: A Shared Framework for Reducing Recidivism and Promoting Recovery. Council of State Governments Justice Center (2012). Available online at: https://bja.ojp.gov/sites/g/files/xyckuh186/files/Publications/CSG_Behavioral_Framework.pdf

[16] Branje SJT Hale WW Frijns T Meeus WHJ. Longitudinal associations between perceived parent-child relationship quality and depressive symptoms in adolescence. J Abnorm Child Psychol. (2010) 38:751–63. 10.1007/s10802-010-9401-6 20217211 PMC2902740

[17] Christian S. Children of Incarcerated Parents. Washington DC: National Conference on State Legislatures (2009).

[18] Guastaferro K Guastaferro WP Brown JR Holleran D Whitaker DJ. Drug court as an intervention point to affect the well-being of families of parents with substance use disorders. Subst Use Misuse. (2020) 55:1068–78. 10.1080/10826084.2020.1726394 32091939 PMC7180132

[19] Goodman KL De Los Reyes A Bradshaw CP. Understanding and using informants' reporting discrepancies of youth victimization: a conceptual model and recommendations for research. Clin Child Fam Psychol Rev. (2010) 13:366–83. 10.1007/s10567-010-0076-x 20799062

[20] Fung JJ Lau AS. Factors associated with parent-child (Dis)agreement on child behavior and parenting problems in chinese immigrant families. J Clin Child Adolesc Psychol. (2010) 39:314–27. 10.1080/15374411003691693 20419573 PMC7055478

[21] Leung JTY Shek DTL. Parent-adolescent discrepancies in perceived parenting characteristics and adolescent developmental outcomes in poor Chinese families. J Child Fam Stud. (2014) 23:200–13. 10.1007/s10826-013-9775-5 24482569 PMC3890555

[22] De Los Reyes A. Introduction to the special section: more than measurement error: discovering meaning behind informant discrepancies in clinical assessments of children and adolescents. J Clin Child Adolesc Psychol. (2011) 40:1–9. 10.1080/15374416.2011.533405 21229439

[23] Cooley DT Jackson Y. Informant discrepancies in child maltreatment reporting: a systematic review. Child Maltreat. (2020) 2020:1077559520966387. 10.1177/1077559520966387 33054358

[24] Cho B Jackson Y. Self-reported and case file maltreatment: relations to psychosocial outcomes for youth in foster care. Child Youth Serv Rev. (2016) 69:241–7. 10.1016/j.childyouth.2016.08.013

[25] Everson MD Smith JB Hussey JM English D Litrownik AJ Dubowitz H. Concordance between adolescent reports of childhood abuse and child protective service determinations in an at-risk sample of young adolescents. Child Maltreat. (2008) 13:14–26. 10.1177/1077559507307837 18174345

[26] Havlicek J Courtney ME. Maltreatment histories of aging out foster youth: a comparison of official investigated reports and self-reports of maltreatment prior to and during out-of-home care. Child Abuse Negl. (2016) 52:110–22. 10.1016/j.chiabu.2015.12.006 26724824

[27] Kobulsky JM Kepple NJ Holmes MR Hussey DL. Concordance of parent-and child-reported physical abuse following child protective services investigation. Child Maltreat. (2017) 22:24–33. 10.1177/1077559516673156 27742847

[28] Negriff S Schneiderman JU Trickett PK. Concordance between self-reported childhood maltreatment versus case record reviews for child welfare-affiliated adolescents: prevalence rates and associations with outcomes. Child Maltreat. (2017) 22:34–44. 10.1177/1077559516674596 27777329 PMC5353974

[29] Korelitz KE Garber J. Congruence of parents' and children's perceptions of parenting: a meta-analysis. J Youth Adolesc. (2016) 45:1973–95. 10.1007/s10964-016-0524-0 27380467 PMC5222679

[30] Abar CC Jackson KM Colby SM Barnett NP. Parent-child discrepancies in reports of parental monitoring and their relationship to adolescent alcohol-related behaviors. J Youth Adolesc. (2015) 44:1688–701. 10.1007/s10964-014-0143-6 24964878 PMC4511723

[31] Dimler LM Natsuaki MN Hastings PD Zahn-Waxler C Klimes-Dougan B. Parenting effects are in the eye of the beholder: parent-adolescent differences in perceptions affects adolescent problem behaviors. J Youth Adolesc. (2017) 46:1076–88. 10.1007/s10964-016-0612-1 27848126

[32] Kapetanovic S Boson K. Discrepancies in parents' and adolescents' reports on parent-adolescent communication and associations to adolescents' psychological health. Curr Psychol. (2020). 10.1007/s12144-020-00911-0. [Epub ahead of print].

[33] Maurizi LK Gershoff ET Aber JL. Item-level discordance in parent and adolescent reports of parenting behavior and its implications for adolescents' mental health and relationships with their parents. J Youth Adolesc. (2012) 41:1035–52. 10.1007/s10964-011-9741-8 22258760 PMC7988820

[34] De Los Reyes A Ohannessian CM Racz SJ. Discrepancies between adolescent and parent reports about family relationships. Child Dev Perspect. (2019) 13:53–8. 10.1111/cdep.12306

[35] Ondersma SJ Chaffin MJ Mullins SM LeBreton JM. A brief form of the child abuse potential inventory: development and validation. J Clin Child Adolesc Psychol. (2005) 34:301–11. 10.1207/s15374424jccp3402_9 15901230

[36] Derogatis LR Melisaratos N. The brief symptom inventory: an introductory report. Psychol Med. (1983) 13:595–605. 10.1017/S0033291700048017 6622612

[37] Shelton KK Frick PJ Wootton J. Assessment of parenting practices in families of elementary school-age children. J Clin Child Psychol. (1996) 25:317–29. 10.1207/s15374424jccp2503_8

[38] Frick PJ Christian RE Wootton JM. Age trends in the association between parenting practices and conduct problems. Behav Modif. (1999) 23:106–28. 10.1177/0145445599231005

[39] Reynolds CR Kamphaus RW. Behavior Assessment System for Children (2nd Edn.). American Guidance Service (2004).

[40] SAS Institute Inc. SAS/ACCESS® 9.4. SAS Institute Inc (2013).

[41] Flight L Julious SA. The disagreeable behaviour of the kappa statistic. Pharm Stat. (2015) 14:74–8. 10.1002/pst.1659 25470361

[42] Landis JR Koch GG. The measurement of observer agreement for categorical data. Biometrics. (1977) 33:159–74. 10.2307/2529310 843571

[43] Babyak MA. What you see may not be what you get: a brief, nontechnical introduction to overfitting in regression-type models. Psychosom Med. (2004) 66:411–21. 10.1097/00006842-200405000-00021 15184705

[44] Schneider W MacKenzie M Waldfogel J Brooks-Gunn J. Parent and child reporting of corporal punishment: new evidence from the fragile families and child wellbeing study. Child Indic Res. (2015) 8:347–58. 10.1007/s12187-014-9258-2 28386302 PMC5380381

[45] Weymouth BB Buehler C Zhou N Henson RA. A meta-analysis of parent-adolescent conflict: disagreement, hostility, youth maladjustment. J Fam Theory Rev. (2016) 8:95–112. 10.1111/jftr.12126

[46] Rusby JC Light JM Crowley R Westling E. Influence of parent-youth relationship, parental monitoring, and parent substance use on adolescent substance use onset. J Fam Psychol. (2018) 32:310–20. 10.1037/fam,0000350 29300096 PMC5920742

